# Response to correspondence from Hays and colleagues concerning our paper entitled, use of the KDQOL-36™ for assessment of health-related quality of life among dialysis patients in the United States

**DOI:** 10.1186/s12882-019-1608-3

**Published:** 2019-12-03

**Authors:** Dena E. Cohen, Andrew Lee, Scott Sibbel, Deborah Benner, Steven M. Brunelli, Francesca Tentori

**Affiliations:** 1 DaVita Clinical Research, 825 S 8th St, Minneapolis, MN 55404 USA; 2 DaVita, Inc, 2000 16th St, Denver, CO 80202 USA

**Keywords:** Renal disease, Health-related quality of life, KDQOL-36TM

## Abstract

In their correspondence, Hays et al. raise two main critiques of our recently published article entitled “Use of the KDQOL-36™ for assessment of health-related quality of life among dialysis patients in the United States.” First, Hays et al. expressed concerns regarding the comparison of mean scores on five Kidney Disease Quality of Life (KDQOL) subscales, given that the Physical Component Summary (PCS) and Mental Component Summary (MCS) are scored on a different numeric scale compared to the other three subscales. Second, Hays et al. note that the correlations reported in our manuscript between the general health perceptions item (“In general, would you say your health is excellent, very good, good, fair, or poor”) and the 5 KDQOL subscales were inconsistent with findings derived from other KDQOL datasets. Here, we respond to these two critiques.

We appreciate the detailed review and commentary on our manuscript [1] provided by Hays et al. [2]. We agree with their observation that, because the physical component summary (PCS) and Symptoms and Problems of Kidney Disease (SPKD) subscales are scored on different scales, direct comparison of the scores is not appropriate. For this reason, we were careful to note that the mean scores that we observed (36.6 vs 73.0 respectively) convey different impressions about patients’ perceptions of their health, but we did not make any attempt to interpret the specific meaning of the numeric difference. We agree with Hays et al. that, in the future, consistent use of a single scoring method for all KDQOL subscales would greatly facilitate interpretation and contextualization of these scores.

Hays et al. raised an important question with regard to the correlation of the general health rating item and the 5 subscale scores as reported in our manuscript. Upon review, we have determined that indeed the correlations reported in our manuscript were based on analyses that included a coding error. This was an honest mistake that was not captured at the time of submission, and we thank Hays et al. for bringing this to our attention. Upon reanalysis, we find correlations that are broadly consistent with those reported by Hays et al., and our manuscript will be corrected to reflect these findings. Importantly, we have conducted a detailed review of the entire dataset and analysis underlying the remaining results presented in the manuscript and found no additional errors. Thus, we stand by all of the other results and conclusions as originally presented.

Improving health-related quality of life, and the tools for its evaluation, remains a top priority for the entire end-stage kidney disease (ESKD) community. Like Hays et al., we believe that successful efforts in this regard will require the combined efforts of patients, providers, and researchers working in both industry and academia. We remain fully committed to participation in these collaborative endeavors.

## Data Availability

The datasets generated and/or analyzed during the current study are not publicly available due to the fact that they are derived from the proprietary database of a large dialysis organization.

## Abbreviations

ESKD: End-stage kidney disease
KDQOL: Kidney disease quality of life
PCS: Physical component summary
SPKD: Symptoms and problems of kidney disease
